# Risk Factors and Precipitants of Severe Disability Among Community-Living Older Persons

**DOI:** 10.1001/jamanetworkopen.2020.6021

**Published:** 2020-06-02

**Authors:** Thomas M. Gill, Ling Han, Evelyne A. Gahbauer, Linda Leo-Summers, Terrence E. Murphy

**Affiliations:** 1 Department of Internal Medicine, Yale School of Medicine, New Haven, Connecticut

## Abstract

**Importance:**

Severe disability greatly diminishes quality of life and often leads to a protracted period of long-term care or death, yet the processes underlying severe disability have not been fully evaluated.

**Objective:**

To evaluate potential risk factors and precipitants associated with severe disability that develops progressively (during ≥2 months) vs catastrophically (from 1 month to the next).

**Design, Setting, and Participants:**

Prospective cohort study conducted in greater New Haven, Connecticut, from March 1998 to December 2016, with 754 nondisabled community-living persons aged 70 years or older. Data analysis was conducted from November 2018 to May 2019.

**Main Outcomes and Measures:**

Candidate risk factors were assessed every 18 months. Functional status and potential precipitants, including illnesses or injuries leading to hospitalization, emergency department visit, or restricted activity, were assessed each month. Severe disability was defined as the need for personal assistance with at least 3 of 4 essential activities of daily living. The analysis was based on person-months within 18-month intervals.

**Results:**

The mean (SD) age for the 754 participants was 78.4 (5.3) years, 487 (64.6%) were women, and 683 (90.5%) were non-Hispanic white participants. The incidence of progressive and catastrophic severe disability was 3.5% and 9.7%, respectively, based on 3550 intervals. In multivariable analysis, 6 risk factors were independently associated with progressive disability (≥85 years: hazard ratio [HR], 1.6; 95% CI, 1.1-2.4; hearing impairment: HR, 1.7; 95% CI, 1.0-2.8; frailty: HR, 2.4; 95% CI, 1.6-3.7; cognitive impairment: HR, 2.0; 95% CI, 1.3-3.1; low functional self-efficacy: HR, 1.8; 95% CI, 1.2-2.8; low peak flow: HR, 1.7; 95% CI, 1.2-2.4), and 4 were independently associated with catastrophic disability (visual impairment: HR, 1.4; 95% CI, 1.1-1.8; hearing impairment: HR, 1.3; 95% CI, 1.0-1.7; poor physical performance: HR, 1.8; 95% CI, 1.3-2.5; low peak flow: HR, 1.3; 95% CI, 1.0-1.7). The associations of the precipitants were much more pronounced than those of the risk factors, with HRs as high as 321.4 (95% CI, 194.5-531.0) for hospitalization and catastrophic disability and 48.3 (95% CI, 31.0%-75.4%) for hospitalization and progressive disability. Elimination of an intervening hospitalization was associated with a decrease in the risk of progressive and catastrophic severe disability of 3.0% (95% CI, 3.0%-3.1%) and 12.3% (95% CI, 12.1%-12.5%), respectively. Risk differences were 0.6% (95% CI, 0.6%-0.6%) and 1.3% (95% CI, 1.3%-1.4%) for emergency department visit and 0.1% (95% CI, 0.1%-0.2%) and 0.4% (95% CI, 0.4%-0.4%) for restricted activity, and ranged from 0.1% (95% CI, 0.1%-0.1%) to 0.3% (95% CI, 0.3%-0.3%) for the independent risk factors, for progressive and catastrophic disability, respectively.

**Conclusions and Relevance:**

The findings of this study suggest that whether it develops progressively or catastrophically, severe disability among older community-living adults arises most commonly in the setting of an intervening illness or injury. To reduce the burden of severe disability, more aggressive efforts will be needed to prevent and manage intervening illnesses or injuries and to facilitate recovery after these debilitating events.

## Key Points

**Question:**

What are the relative contributions of traditional risk factors and intervening illnesses or injuries (ie, precipitants) associated with severe disability that develops progressively vs catastrophically?

**Findings:**

In this cohort study of 754 nondisabled community-living persons aged 70 years or older who were followed longitudinally for nearly 19 years, the associations of intervening illnesses or injuries with severe disability were much more pronounced than those of the risk factors, with hazard ratios up to 20-fold greater for progressive disability and 177-fold greater for catastrophic disability.

**Meaning:**

The findings of this study suggest that to reduce the burden of severe disability, more aggressive efforts will be needed to prevent and manage intervening illnesses or injuries and to facilitate recovery after these debilitating events.

## Teaser

This cohort study evaluates potential risk factors and precipitants associated with severe disability that develops progressively (during ≥2 months) vs catastrophically (from 1 month to the next) in older adults.

## Introduction

The onset of severe disability, defined as the need for personal assistance with 3 or more essential activities of daily living, greatly diminishes quality of life and often leads to a protracted period of long-term care or death.^1,2,3^ These episodes of severe disability are expensive,^4,5,6^ and they are highly stressful to both patients and their caregivers.^7,8^

Previous research, based on annual assessments of functional status, has attempted to distinguish between severe disability that develops progressively through an intermediary stage of mild disability and severe disability that develops abruptly or catastrophically.^9,10,11,12^ This work has provided important insights into potential risk factors for severe disability,^13^ including its 2 major subtypes,^11,12^ and has suggested that severe disability, particularly the catastrophic subtype, is commonly precipitated by an intervening illness or injury leading to hospitalization.^10^ However, the applicability of these findings to clinical practice may be limited for at least 3 reasons. First, annual assessments cannot fully account for the dynamic nature of disability, which is often characterized by frequent transitions in functional status during much shorter periods.^14,15^ Second, temporal precedence between a potential precipitant and functional transition is difficult to establish when assessment intervals are as long as a year. Third, increasing evidence suggests that disability may be precipitated or worsened by less serious illnesses and injuries that do not lead to hospitalization.^16,17,18,19^

To address these limitations, we used high-quality data from a unique longitudinal study of community-living older persons that includes monthly assessments of functional status and potential precipitants, including emergency department visits, episodes of restricted activity, hospitalizations, and a large array of potential risk factors that were assessed every 18 months for nearly 19 years. Our objective was to evaluate potential risk factors and precipitants associated with severe disability that develops progressively vs catastrophically. The results of this study may inform the development of evidence-based interventions to prevent the onset of severe disability and, hence, to reduce the need for long-term care and associated increases in the cost of care.

## Methods

### Study Population

Participants were members of the Precipitating Events Project, an ongoing longitudinal study of 754 community-living persons aged 70 years or older who were initially nondisabled in their essential activities of daily living.^20,21^ Potential participants were members of a large health plan and were excluded for significant cognitive impairment with no available proxy,^22^ life expectancy less than 12 months, plans to move out of the area, or inability to speak English. Only 126 of 2735 individuals (4.6%) refused screening, and 754 of 1002 (75.2%) who were eligible agreed to participate and were enrolled from March 1998 to October 1999. The study was approved by the Yale Human Investigation Committee, and all participants provided verbal informed consent. This study followed the Strengthening the Reporting of Observational Studies in Epidemiology (STROBE) reporting guideline.

### Data Collection

Data on the candidate risk factors were collected during comprehensive home-based assessments, whereas those on disability and potential precipitants were obtained from monthly telephone interviews and Medicare claims. The comprehensive assessments were completed by trained nurse researchers at baseline and every 18 months (completion rate, approximately 95%), whereas the telephone interviews were completed by a separate team of researchers through December 2016 (completion rate, approximately 99%). For participants who had significant cognitive impairment or were otherwise unavailable, we interviewed a proxy informant using a rigorous protocol.^22^ Participants were asked to identify their race/ethnicity primarily for descriptive purposes. Deaths were ascertained by review of obituaries, from an informant during a subsequent interview, or both.

### Candidate Risk Factors

In addition to demographic factors, we considered risk factors from 4 domains found to be associated with disability in previous studies.^23^ The health-related factors included 9 self-reported physician-diagnosed chronic conditions,^15^ vision,^24^ hearing,^25^ and frailty.^26^ The cognitive-psychosocial factors included cognitive status,^27^ depressive symptoms,^28^ functional self-efficacy,^29^ and social support.^30^ The behavioral factors included smoking and body mass index.^31^

The physical capacity factors included a modified version of the Short Physical Performance Battery.^32,33^ The other physical capacity factors included nondominant limb, upper and lower extremity muscle strength,^33^ manual dexterity,^34^ gross motor coordination,^34^ and peak expiratory flow.^35^ To enhance clinical interpretability and facilitate calculation of risk differences, the risk factors were dichotomized with accepted cut points. Additional operational details are provided in Table 1.^26,32,33,36,37^

**Table 1.** Candidate Risk Factors and Their Bivariate Associations With Time to Onset of Progressive and Catastrophic Severe Disability^a^

Characteristic	Measurement details	No severe disability, No. (%) (n = 3081)^b^	Progressive severe disability (n = 125)		Catastrophic severe disability (n = 344)	
			No. (%)	Hazard ratio (95% CI)	No. (%)	Hazard ratio (95% CI)
Demographic						
≥85 y	NA	888 (28.8)	67 (54.0)	2.8 (2.0-4.0)	155 (45.1)	2.0 (1.6-2.5)
Women	NA	2000 (64.9)	80 (64.0)	1.0 (0.7-1.4)	216 (62.8)	0.9 (0.7-1.2)
Non-Hispanic white	NA	2763 (89.7)	115 (92.0)	1.3 (0.7-2.5)	303 (88.1)	0.9 (0.6-1.2)
Lives alone	NA	1337 (43.4)	66 (52.8)	1.5 (1.0-2.1)	164 (47.7)	1.2 (1.0-1.5)
Education <12 y	NA	901 (29.2)	41 (32.8)	1.2 (0.8-1.7)	112 (32.6)	1.2 (0.9-1.5)
Health related						
>2 Chronic conditions^c^	Of 9 self-reported, physician-diagnosed conditions	954 (31.0)	54 (43.2)	1.7 (1.2-2.3)	170 (49.4)	2.1 (1.7-2.7)
Visual impairment, %	>26, assessed with a Jaeger card	543 (17.6)	35 (28.0)	1.7 (1.2-2.5)	114 (33.1)	2.2 (1.8-2.8)
Hearing impairment	≥2 of 4 tones missed^d^	1920 (62.3)	103 (82.4)	2.8 (1.7-4.4)	259 (75.3)	1.8 (1.4-2.3)
Frailty	Fried phenotype^e^	781 (23.4)	86 (68.8)	6.2 (4.3-8.9)	182 (52.9)	3.1 (2.5-3.9)
Cognitive-psychosocial score						
Cognitive impairment	Folstein MMSE <24	330 (10.7)	33 (26.4)	2.9 (1.9-4.3)	68 (19.8)	2.0 (1.5-2.6)
Depressive symptoms	CES-D ≥20	435 (14.1)	28 (22.4)	1.7 (1.1-2.6)	77 (22.4)	1.7 (1.3-2.2)
Low functional self-efficacy^f^	≤27	964 (31.3)	86 (68.8)	4.6 (3.2-6.8)	195 (55.7)	2.8 (2.2-3.4)
Low social support^g^	MOS ≤18	694 (22.5)	32 (25.6)	1.2 (0.8-1.8)	82 (23.8)	1.1 (0.8-1.4)
Behavioral						
Current smoker	NA	177 (5.7)	4 (3.2)	0.6 (0.2-1.4)	19 (5.5)	1.0 (0.6-1.6)
Obesity	Body mass index ≥30^h^	628 (20.4)	20 (16.0)	0.7 (0.5-1.2)	65 (18.9)	0.9 (0.7-1.2)
Physical capacity						
Low SPPB score^i^	<7	1426 (46.3)	104 (83.2)	5.5 (3.5-8.7)	264 (76.7)	3.7 (2.8-4.7)
Muscle weakness						
Upper extremity, kg	Shoulder flexion <11.5 (women), <16.1 (men)^j^	1712 (55.6)	101 (80.8)	3.4 (2.2-5.2)	242 (70.4)	1.9 (1.5-2.4)
Lower extremity, kg	Hip abduction <7.9 (women), <12.6 (men)^j^	1874 (60.8)	104 (83.2)	3.1 (1.9-4.9)	267 (77.6)	2.2 (1.7-2.8)
Manual dexterity, s	Worst quartile, 9-hole pegboard test^k^	1076 (34.9)	80 (64.0)	3.2 (2.3-4.6)	194 (56.4)	2.3 (1.9-2.9)
Gross motor coordination, s	Worst quartile, 10 finger taps^k^	896 (29.1)	60 (48.0)	2.2 (1.5-3.1)	161 (46.8)	2.1 (1.7-2.6)
Low peak flow, %^l^	<10 standardized residual percentile	628 (20.4)	46 (36.8)	2.2 (1.5-3.2)	113 (32.9)	1.9 (1.5-2.3)

Abbreviations: CES-D, Center for Epidemiological Studies–Depression Scale; MMSE, Mini-Mental State Examination; MOS, Medical Outcomes Study Social Support Scale; NA, not applicable; SPPB, Short Physical Performance Battery.

^a^ Characteristics were assessed at the beginning of each 18-month person-interval. The number of observations (ie, person-intervals) is provided for each of the 3 disability groups. These observations were identified from 696 (not severe disability), 124 (progressive severe disability), and 298 (catastrophic severe disability) participants. The mean (SD) number of observations per participant was 4.7 (3.1). Among the 298 participants with catastrophic severe disability, 255 (85.6%) had 1 episode, 40 (13.4%) had 2 episodes, and 3 (1.0%) had 3 episodes.

^b^ Observations (18-month intervals) classified as not severe disability could include months with mild disability as long as severe disability did not develop during the interval.

^c^ Chronic conditions included hypertension, myocardial infarction, heart failure, stroke, diabetes, arthritis, hip fracture, chronic lung disease, and cancer (other than minor skin cancers); the cut point was selected according to the American Geriatrics Society Expert Panel on the Care of Older Adults With Multimorbidity.^36^

^d^ Based on 1000- and 2000-Hz measurements for the left and right ears.

^e^ Based on the 5 following standard criteria: weight loss, exhaustion, low physical activity, muscle weakness, and slow walking speed.^26^

^f^ Maximum score is 40, based on level of confidence in performing the 10 following activities (each scored 0 to 4): dressing, bathing and showering, transferring, going up and down stairs, walking around the neighborhood, housecleaning, preparing simple meals, simple shopping, reaching into cabinets or closets, and hurrying to answer the telephone. Cut point demarcates the worst quartile, based on the first 356 enrolled participants who had been selected randomly from the source population.

^g^ Cut point demarcates the worst quartile, based on the first 356 enrolled participants who had been selected randomly from the source population.

^h^ Body mass index calculated as weight in kilograms divided by height in meters squared, and category was based on established cut point.^37^

^i^ Cut point denotes low physical capacity.^32^

^j^ Assessed with handheld Chatillon MSE 100 dynamometer; cut points demarcate the worst sex-specific quartile for nondominant limb, based on the first 356 enrolled participants who had been selected randomly from the source population.^33^

^k^ Because standard cut points have not been established, quartile scores were calculated according to the first 356 enrolled participants and subsequently applied to the entire cohort.

^l^ Based on previously validated cut point.^33^

### Potential Precipitants

Potential precipitants included intervening illnesses or injuries leading to hospitalization, emergency department (ED) visit, or restricted activity. The primary source of information on hospitalizations and ED visits was linked Medicare claims data, which were available for nearly all hospitalizations and for ED visits among fee-for-service participants.^15^ For periods when participants had managed Medicare, hospitalizations were ascertained with Medicare Provider Analysis and Review files, whereas information on ED visits and some hospitalizations was obtained during the monthly interviews. Participants were asked whether they had visited an ED or stayed overnight in a hospital since the last interview. The accuracy of this information was high.^16,26^ Hospitalizations were subsequently classified as critical illness, major surgery, or neither, as previously described.^38,39^ For descriptive purposes, the reasons for ED visits and hospitalizations were subsequently grouped into distinct diagnostic categories according to revised versions of a previously described protocol.^10,16,17^

In accordance with a standardized protocol with high reliability, participants were asked 2 questions related to restricted activity to ascertain less potent precipitants.^20^ Participants who answered yes to 1 or both questions were asked to identify the reason(s) for their restricted activity. We have previously shown that older persons usually attribute their restricted activity to several concurrent health-related problems.^20^

The potential precipitants were organized into 3 mutually exclusive hierarchic categories: hospitalization, ED visit but no hospitalization, and restricted activity but no hospitalization or ED visit.

### Disability Assessments

Complete details regarding the assessment of disability, including reliability and accuracy, are provided elsewhere.^21,22^ Each month, participants were asked, “At the present time, do you need help from another person to [complete the task]?” for the 4 following essential activities: bathing, dressing, walking, and transferring. Disability was operationalized as the need for personal assistance, and the severity of disability was denoted by the number of disabled activities (0-4) in a specific month. Disability in 1 to 2 activities was considered mild, whereas disability in 3 to 4 activities was considered severe.^14,21^ For participants with major cognitive impairment, the monthly interviews were completed with a designated proxy.

### Statistical Analysis

The analysis was based on person-months within 18-month intervals. Participants could contribute more than one 18-month interval (ie, observation) to the analysis, but they had to be nondisabled and living in the community at the start of each interval. Of the 4702 person-intervals through December 2016, 3550 (75.5%) were eligible. For each observation, we evaluated time to first occurrence of severe disability from the start of the interval. Severe disability was classified as catastrophic if it developed from 1 month to the next and progressive if it developed during 2 or more months through an intermediary stage of mild disability.^9^ In accordance with previous work,^40^ we evaluated the association between the potential precipitants and onset of severe disability (progressive and catastrophic) monthly.

The characteristics of the candidate risk factors, assessed at the start of each 18-month interval, were summarized according to type of severe disability per interval (ie, progressive, catastrophic, or neither). For each of these 3 groups, exposure to the potential precipitants was calculated per 100 person-months with an intercept-only Poisson model with generalized estimating equations and autoregressive correlation.

We used pooled logistic regression with a complementary log-log link and generalized estimating equations with robust standard errors based on an autoregressive correlation structure to evaluate the bivariate and multivariable relationships between candidate risk factors and potential precipitants, respectively, and discrete time (in months from the start of the interval) to first occurrence of either of the 2 severe disability outcomes, referred to also as progressive and catastrophic disability. This analytic strategy permits the calculation of hazard ratios.^41,42^ For the multivariable analyses, we used backward selection with *P* < .20. The 18-month interval was included as a count variable to account for calendar time. As previously described,^43,44^ a series of sensitivity analyses was performed to assess for potential bias caused by the competing risk of death. To enhance clinical interpretability, we calculated risk differences with bootstrapped CIs for each of the statistically significant risk factors and precipitants from the final multivariable models.^45^ The risk difference denotes how much the population-level risk of an outcome would be reduced if the relevant factor were removed. We also ran an alternative form of the final multivariable model that subcategorized hospitalization as critical illness,^38^ major surgery^39^ (exclusive of critical illness), or other.

The amount of missing data for the candidate risk factors ranged from 0.3% for smoking to 8.1% for peak flow, with the exception of upper and lower extremity muscle strength, for which 10.2% and 9.7% of the observations were missing, respectively. To address these missing data, we used sequential Markov chain Monte Carlo imputation for multivariate normal data.^46^ Missing data were not imputed for the potential precipitants (0%, 0.4%, and 0% of the observations for hospitalization, ED visits, and restricted activity, respectively).

All analyses were conducted with SAS version 9.4 (SAS Institute). Statistical significance was defined as a 2-tailed *P* < .05.

## Results

The mean (SD) age for the 754 participants was 78.4 (5.3) years, 487 (64.6%) were women, and 683 (90.5%) were non-Hispanic white participants. Severe disability developed in 469 (13.2%) of the 3550 person-intervals. The incidence of catastrophic disability (9.7%) was considerably greater than that of progressive disability (3.5%), whereas the mean (SD) time to develop progressive disability (ie, 10.9 [4.9] months) was modestly longer than that of catastrophic disability (9.0 [5.3] months). The characteristics of the 3 disability groups are provided in Table 1. With few exceptions, the most favorable characteristics were observed for the group without severe disability, whereas the least favorable were observed for the progressive severe disability group.

The bivariate associations of the candidate risk factors with the 2 disability outcomes are also provided in Table 1. For each outcome, the strongest associations were observed for frailty (progressive severe disability: hazard ratio [HR], 6.2; 95% CI, 4.3-8.9; catastrophic severe disability: HR, 3.1; 95% CI, 2.5-3.9), low Short Physical Performance Battery score (progressive severe disability: HR, 5.5; 95% CI, 3.5-8.7; catastrophic severe disability: HR, 3.7; 95% CI, 2.8-4.7), and low functional self-efficacy (progressive severe disability: HR, 4.6; 95% CI, 3.2-6.8; catastrophic severe disability: HR, 2.8; 95% CI, 2.2-3.4). No other factor achieved an unadjusted hazard ratio of 3 or greater for catastrophic disability, and only 3 achieved this level for progressive disability: upper extremity muscle weakness (HR, 3.4; 95% CI, 2.2-5.2), lower extremity muscle weakness (HR, 3.1; 95% CI, 1.9-4.9), and low manual dexterity (HR, 3.2; 95% CI, 2.3-4.6).

As shown in Table 2, the rates of hospitalizations and ED visits were substantially lower in the group without severe disability than in the groups with progressive severe disability and with catastrophic severe disability (hospitalization mean [SD] exposure rate: 2.2 [0.1] vs 10.6 [0.9] vs 10.8 [0.6]; ED visit mean [SD] exposure rate: 1.9 [0.1] vs 5.6 [0.8] vs 3.4 [0.3]), whereas the rate of restricted activity was only modestly lower (mean [SD] exposure rate, 11.5 [0.4] vs 12.3 [1.2] vs 14.3 [0.9]). The bivariate associations between the potential precipitants and 2 disability outcomes were very large, especially for hospitalization (progressive severe disability: HR, 62.9; 95% CI, 40.9-96.7; catastrophic severe disability: HR, 375.3; 95% CI, 228.4-616.5). For each precipitant, the associations were much stronger for catastrophic than progressive severe disability (eg, ED visits: HR, 38.3 [95% CI, 19.2-76.2] vs 12.4 [95% CI, 5.9-26.0]). The reasons for hospitalizations, ED visits, and restricted activity are provided in eTables 1, 2, and 3 in the Supplement. For each disability group, the most common reasons for hospitalization were cardiac (without severe disability: 342 of 1400 [24.5%]; progressive severe disability: 27 of 172 [15.6%]; catastrophic severe disability: 60 of 395 [15.2%]) and infection (without severe disability: 175 [12.5%]; progressive severe disability: 35 [20.2%]; catastrophic severe disability: 67 [17.0%]); for ED visits, musculoskeletal (without severe disability: 279 of 1005 [27.8%]; progressive severe disability: 28 of 74 [37.8%]; catastrophic severe disability: 24 of 89 [27.0%]); and for restricted activity, fatigue (mean [SD] episodes per 100 person-months without severe disability: 61.3 [0.5]; progressive severe disability: 72.8 [0.4]; catastrophic severe disability: 71.0 [0.5]) and pain or stiffness in joints (without severe disability: 38.8 [0.5]; progressive severe disability: 46.2 [0.5]; catastrophic severe disability: 49.0 [0.5]). Differences across the 3 groups were most pronounced for hospitalizations for fall-related injury (without severe disability: 54 [3.9%]; progressive severe disability: 12 [7.0%]; catastrophic severe disability: 52 [13.2%]); for ED visits, cardiac (without severe disability: 105 [10.5%]; progressive severe disability: 10 [13.5%]; catastrophic severe disability: 2 [2.3%]), falls or mobility problems (without severe disability: 48 [4.8%]; progressive severe disability: 11 [14.9%]; catastrophic severe disability: 11 [12.4%]); for restricted activity, dizziness or unsteadiness on feet (mean [SD] episodes per 100 person-months without severe disability: 27.0 [0.4]; progressive severe disability: 45.1 [0.5]; catastrophic severe disability: 34.9 [0.5]).

**Table 2.** Exposure to Potential Precipitants and Bivariate Associations With Time to Onset of Progressive and Catastrophic Severe Disability^a^

Potential precipitant^b^	No severe disability (n = 3081)^c^	Progressive severe disability (n = 125)		Catastrophic severe disability (n = 344)	
	Exposure rate (SD)	Exposure rate (SD)	Hazard ratio (95% CI)^d^	Exposure rate (SD)	Hazard ratio (95% CI)^d^
Hospitalization	2.2 (0.1)	10.6 (0.9)	62.9 (40.9-96.7)	10.8 (0.6)	375.3 (228.4-616.5)
Emergency department visit	1.9 (0.1)	5.6 (0.8)	12.4 (5.9-26.0)	3.4 (0.3)	38.3 (19.2-76.2)
Restricted activity^e^	11.5 (0.4)	12.3 (1.2)	3.7 (2.0-6.7)	14.3 (0.9)	11.4 (6.2-20.8)

^a^ The number of observations (ie, person-intervals) is provided for each of the 3 disability groups. These observations were identified from 696 (not severe disability), 124 (progressive severe disability), and 298 (catastrophic severe disability) participants. The exposure rates are per 100 person-months.

^b^ The 3 potential precipitants are mutually exclusive and hierarchic, as described in the Methods section.

^c^ Observations (18-month intervals) classified as not severe disability could include months with mild disability as long as severe disability did not develop during the interval.

^d^ Values represent the risk of the disability outcome in the setting of the specific precipitant relative to no precipitant.

^e^ To ascertain restricted activity, participants were asked the following 2 questions each month: “Since we last talked on [date of last interview], have you cut down on your usual activities because of an illness, injury, or other problem?” and “Since we last talked on [date of last interview], have you stayed in bed for at least half a day because of an illness, injury, or other problem?”

The Figure provides the multivariable results for the risk factors and precipitants associated with time to onset of progressive and catastrophic severe disability, respectively. In the final models, 6 risk factors were independently associated with progressive disability (≥85 years: HR, 1.6; 95% CI, 1.1-2.4; hearing impairment: HR, 1.7; 95% CI, 1.0-2.8; frailty: HR, 2.4; 95% CI, 1.6-3.7; cognitive impairment: HR, 2.0; 95% CI, 1.3-3.1; low functional self-efficacy: HR, 1.8; 95% CI, 1.2-2.8; low peak flow: HR, 1.7; 95% CI, 1.2-2.4), and 4 were independently associated with catastrophic disability (visual impairment: HR, 1.4; 95% CI, 1.1-1.8; hearing impairment: HR, 1.3; 95% CI, 1.0-1.7; low Short Physical Performance Battery score: HR, 1.8; 95% CI, 1.3-2.5; low peak flow: HR, 1.3; 95% CI, 1.0-1.7). The associations for progressive disability were strongest for frailty and cognitive impairment, whereas those for catastrophic disability were strongest for low Short Physical Performance Battery score and visual impairment. For both types of severe disability, the associations of the precipitants were much more pronounced than those of the risk factors, with the most potent associations observed for hospitalization, followed by ED visit and restricted activity. For example, the adjusted HR was as high as 321.4 (95% CI, 194.5-531.0) for hospitalization and catastrophic disability and 48.3 (95% CI, 31.0%-75.4%) for hospitalization and progressive disability. The results did not change when age was analyzed as a continuous rather than dichotomous variable. In the sensitivity analyses, the associations between the precipitants and 2 severe disability outcomes remained robust to the competing risk of death (eTable 4 and eTable 5 in the Supplement). Severe disability was preceded by a precipitant for 68.0% (85 of 125) of the progressive outcomes and 91.0% (313 of 344) of the catastrophic outcomes.

**Figure.**
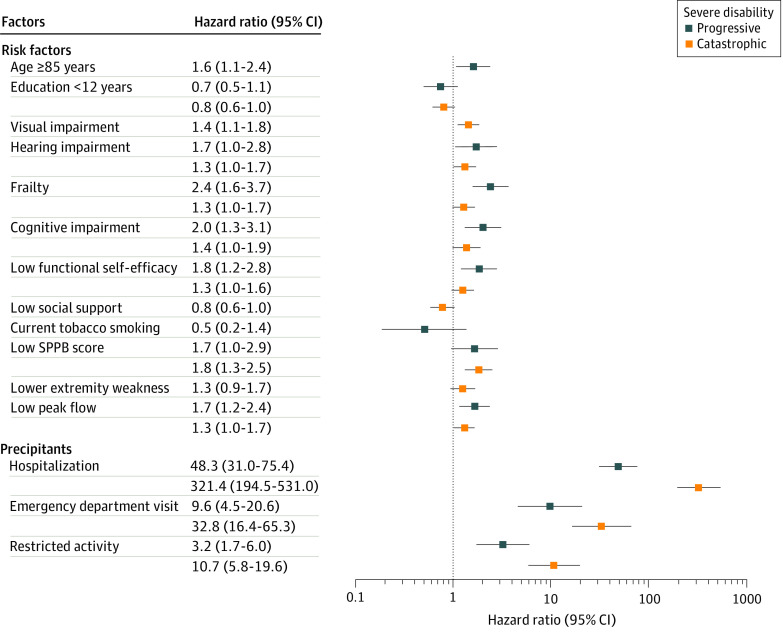
Risk Factors and Precipitants Associated in Multivariable Analysis With Time to Onset of Progressive and Catastrophic Severe Disability Only risk factors with *P* < .20 were retained in the final models, which included the 18-month interval as a count variable to account for calendar time. SPPB indicates Short Physical Performance Battery.

The corresponding results for risk differences are provided in Table 3. Elimination of an intervening hospitalization was associated with a reduction in the risk of progressive and catastrophic severe disability of 3.0% (95% CI, 3.0%-3.1%) and 12.3% (95% CI, 12.1%-12.5%), respectively. The corresponding values were 0.6% (95% CI, 0.6%-0.6%) and 1.3% (95% CI, 1.3%-1.4%) for ED visit and 0.1% (95% CI, 0.1%-0.2%) and 0.4% (95% CI, 0.4%-0.4%) for restricted activity, respectively. The values for the independent risk factors were much lower than those for hospitalization and ED visit and were comparable to those for restricted activity, ranging from 0.1% (95% CI, 0.1%-0.1%) to 0.3% (95% CI, 0.3%-0.3%).

**Table 3.** Risk Differences for Independent Risk Factors and Precipitants^a^

Risk Factor or Precipitant	Risk difference, % (95% CI)	
	Progressive severe disability (n = 125)	Catastrophic severe disability (n = 344)
Risk factors		
≥85 y	0.1 (0.1-0.1)	NA
Visual impairment	NA	0.2 (0.2-0.2)
Hearing impairment	0.1 (0.1-0.1)	0.1 (0.1-0.1)
Frailty	0.2 (0.2-0.2)	NA
Cognitive impairment	0.2 (0.2-0.2)	NA
Low functional self-efficacy	0.1 (0.1-0.1)	NA
Low SPPB score	NA	0.3 (0.3-0.3)
Low peak flow	0.1 (0.1-0.1)	0.1 (0.1-0.2)
Precipitants		
Hospitalization	3.0 (3.0-3.1)	12.3 (12.1-12.5)
Emergency department visit	0.6 (0.6-0.6)	1.3 (1.3-1.4)
Restricted activity	0.1 (0.1-0.2)	0.4 (0.4-0.4)

Abbreviations: NA, not applicable; SPPB, Short Physical Performance Battery.

^a^ The risk difference denotes how much the risk of an outcome would be reduced if the relevant factor were eliminated. To facilitate clinical interpretation and avoid false precision, values are reported to only 1 decimal point.

In the alternative form of the final multivariable model, with no precipitant as the reference group, the adjusted HRs for critical illness, major surgery, and other hospitalizations were, respectively, 133.5 (95% CI, 64.0-278.4), 99.3 (95% CI, 50.6-194.7), and 38.4 (95% CI, 23.9-61.6), respectively, for progressive disability and 1004.6 (95% CI, 563.5-1791.2), 676.8 (95% CI, 394.3-1161.8), and 203.4 (95% CI, 121.9-339.3), respectively, for catastrophic disability.

## Discussion

In this prospective longitudinal study of community-living older persons, we evaluated potential risk factors and precipitants associated with severe disability that develops progressively vs catastrophically. Seven major findings warrant comment. First, the incidence of catastrophic disability was nearly 3 times higher than that of progressive disability. Second, frailty and cognitive impairment were the risk factors most strongly associated with progressive disability, whereas low Short Physical Performance Battery score, an indicator of physical capacity, and visual impairment were the risk factors most strongly associated with catastrophic disability. Third, only 2 risk factors were independently associated with both disability outcomes: hearing impairment and low peak flow. Fourth, associations were considerably stronger for each of the 3 precipitants than any of the risk factors for both outcomes. Fifth, hospitalization was the most potent precipitant, followed by ED visit and restricted activity. Sixth, hospitalizations involving critical illness or major surgery were particularly deleterious. Seventh, risk differences were much lower for the independent risk factors than for hospitalization and ED visit and were comparable to those for restricted activity. These findings suggest that attention to intervening illnesses or injuries will be necessary to prevent the onset of severe disability among older persons and to reduce the resulting need for long-term care.

Prevention of severe disability is important for several reasons. First, the likelihood of recovering independent function is considerably lower for a severe disability than a mild one.^14,21^ Second, the onset of severe disability usually necessitates receipt of long-term care services that are expensive and emotionally taxing.^3,4,5,6,8^ Third, most deaths in older persons are preceded by severe disability.^47^

With few exceptions,^10^ previous studies of severe disability have focused on identifying predisposing risk factors.^13^ Although these factors may help to identify older persons who are more susceptible to developing severe disability, their contribution to this outcome is small relative to that of intervening illnesses or injuries, especially those leading to hospitalization. Our results suggest that the most effective strategies for reducing severe disability in older persons will likely include minimizing preventable illnesses and injuries leading to hospitalization,^48,49,50,51^ decreasing the adverse functional consequences of hospitalization,^52,53,54,55,56^ bolstering restorative therapies after hospitalization,^57,58^ and substituting hospital at home for traditional inpatient care.^59^ Because fall-related injuries were the third leading cause of hospitalizations, resulting in both progressive and catastrophic disability, fall prevention is a particularly attractive strategy for reducing severe disability,^48^ especially if the results of 2 large pragmatic trials are supportive.^60,61^

A unique feature of our study is the availability of data from monthly interviews, which allowed us to more precisely determine the occurrence and severity of disability, more accurately distinguish between progressive and catastrophic disability, and more completely ascertain exposure to potential precipitants (or intervening illnesses and injuries). The frequency of our assessments increases the likelihood that the precipitants preceded the disability outcomes, thereby strengthening temporal precedence and supporting a causal association. We found that severe disability was preceded by a precipitant for nearly all catastrophic outcomes and approximately three-quarters of the progressive outcomes. These values are much higher than those from previous studies that were based on annual assessments of disability and evaluated only hospitalization as a precipitant.^10^ As discussed elsewhere,^17^ the large HRs reflect not only the deleterious effects of the intervening illnesses or injuries but also the low incidence of disability in the absence of such an event. In addition to ascertaining the reason(s) for each precipitant, we also distinguished hospitalizations that included critical illness and major surgery from other admissions, allowing us to demonstrate a dose-response association across these 3 types of hospital-based precipitants.

### Limitations

This study has limitations. We did not ascertain intervening illnesses or injuries that did not lead to hospitalization, ED visit, or restricted activity. A previous study found no relationship between a series of new or worsening conditions, when ascertained without such thresholds, and decline in physical or cognitive performance.^62^ Because this was an observational study, the reported associations, whether assessed as HRs or risk differences, cannot be construed as causal. Even if the associations were causal, the proportion of severe disability that could be prevented through currently available interventions is uncertain. Because our participants were members of a single health plan in a small urban area and because our analyses were restricted to participants who were nondisabled and living in the community, our findings may not be generalizable to older persons in other settings or those with mild disability. However, generalizability depends not only on the choice of the study sample but also on the stability of the sample over time.^63^ A strength of our study is the low attrition rate and nearly complete ascertainment of disability and intervening illnesses or injuries. The generalizability of our findings is also enhanced by our high participation rate, which was greater than 75%.

## Conclusions

The findings of this study suggest that whether it develops progressively or (especially) catastrophically, severe disability among community-living older persons arises most commonly in the setting of an intervening illness or injury. To reduce the burden of severe disability in an aging society, more aggressive efforts will be needed to prevent and manage intervening illnesses or injuries and to facilitate recovery after these debilitating events.

## Notes

Supplement. **eTable 1. **Reasons for Hospitalization According to Type of Severe Disability **eTable 2. **Reasons for Emergency Department Visits According to Type of Severe Disability **eTable 3. **Reasons for Restricted Activity According to Type of Severe Disability **eTable 4. **Sensitivity of Associations Between Precipitants and Progressive Severe Disability to the Competing Risk of Death **eTable 5. **Sensitivity of Associations Between Precipitants and Catastrophic Severe Disability to the Competing Risk of Death
