# Supplementary material for: Risk Factors and Precipitants of Severe Disability Among Community-Living Older Persons
Source: JAMA Netw Open. 2020 Jun 2;3(6):e206021. doi: 10.1001/jamanetworkopen.2020.6021 (PMC7267844; doi:10.1001/jamanetworkopen.2020.6021)
Supplement: Supplement. — eTable 1. Reasons for Hospitalization According to Type of Severe Disability eTable 2. Reasons for Emergency Department Visits According to Type of Severe Disability eTable 3. Reasons for Restricted Activity According to Type of Severe Disability eTable 4. Sensitivity of Associations Between Precipitants and Progressive Severe Disability to the Competing Risk of Death eTable 5. Sensitivity of Associations Between Precipitants and Catastrophic Severe Disability to the Competing Risk of Death [file jamanetwopen-3-e206021-s001.pdf]

Supplementary Online Content

Gill TM, Han L, Gahbauer EA, Leo-Summers L, Murphy TE. Risk factors and precipitants of severe disability among community-living older persons. *JAMA Netw Open*. 2020;3(6):e206021. doi:10.1001/jamanetworkopen.2020.6021

eTable 1. Reasons for Hospitalization According to Type of Severe Disability
eTable 2. Reasons for Emergency Department Visits According to Type of Severe Disability
eTable 3. Reasons for Restricted Activity According to Type of Severe Disability
eTable 4. Sensitivity of Associations Between Precipitants and Progressive Severe Disability to the Competing Risk of Death
eTable 5. Sensitivity of Associations Between Precipitants and Catastrophic Severe Disability to the Competing Risk of Death

This supplementary material has been provided by the authors to give readers additional information about their work.

© 2020 Gill TM et al. *JAMA Network Open.*

eTable 1. Reasons for Hospitalization According to Type of Severe Disability[a]

Reasons for Hospitalization[b]	Not Severe Disability (n=1400)	Progressive Severe Disability (n=172)	Catastrophic Severe Disability (n=395)
		n (%)[c]	
Cardiac	342 (24.5)	27 (15.6)	60 (15.2)
Infection	175 (12.5)	35 (20.2)	67 (17.0)
Stroke	64 (4.6)	3 (1.7)	26 (6.6)
Arthritis	63 (4.5)	7 (4.1)	25 (6.3)
Fall-related injury	54 (3.9)	12 (7.0)	52 (13.2)
Cancer	47 (3.4)	8 (4.7)	31 (7.9)
Gastrointestinal tract bleeding	40 (2.9)	3 (1.7)	11 (2.8)
Other			
Medical	423 (30.2)	59 (34.1)	95 (24.1)
Surgical	168 (12.0)	10 (5.8)	23 (5.8)
Other[d]	15 (1.1)	6 (3.5)	4 (1.0)

Abbreviations: SD, standard deviation

[a]The number of hospitalizations are provided for each of the three disability groups. These hospitalizations were identified from 472 (not severe disability), 97 (progressive severe disability), and 257 (catastrophic severe disability) participants. Reasons are missing for 9 (0.6%), 2 (1.2%), and 1 (0.3%) hospitalizations in the not severe, progressive severe, and catastrophic severe disability groups, respectively.

[b]Grouped into distinct diagnostic categories, as described in the “Methods” section, and presented in order of highest to lowest rates in the not severe disability group.

[c]For each calculation, the numerator includes the number of admissions during the follow-up period for the specific reason, while the denominator includes the number of admissions for all reasons combined.

[d]Includes psychiatric admissions and responses that could not otherwise be categorized.

© 2020 Gill TM et al. *JAMA Network Open.*

eTable 2. Reasons for Emergency Department Visits According to Type of Severe Disability[a]

Reasons for Emergency Department Visits[b]	Not Severe Disability (n=1005)	Progressive Severe Disability (n=74)	Catastrophic Severe Disability (n=89)
		n (%)[c]	
Musculoskeletal	279 (27.8)	28 (37.8)	24 (27.0)
Cardiac	105 (10.5)	10 (13.5)	2 (2.3)
Gastrointestinal	94 (9.4)	3 (4.1)	8 (9.0)
Infection	86 (8.6)	3 (4.1)	3 (3.4)
Head and neck	54 (5.4)	2 (2.7)	6 (6.7)
Falls/mobility problems	48 (4.8)	11 (14.9)	11 (12.4)
Renal/genitourinary	47 (4.7)	2 (2.7)	2 (2.3)
Pulmonary	33 (3.3)	4 (5.4)	2 (2.3)
Neurologic	30 (3.0)	3 (4.1)	3 (3.4)
Dermatologic	28 (2.8)	0	5 (5.6)
Toxic/environmental	6 (0.6)	0	0
Psychiatric	5 (0.5)	1 (1.4)	0
Other medical[d]	156 (15.5)	6 (8.1)	18 (20.2)

Abbreviations: SD, standard deviation

[a]The number of emergency department visits that did not lead to hospitalization are provided for each of the three disability groups. These visits were identified from 371 (not severe disability), 43 (progressive severe disability), and 72 (catastrophic severe disability) participants. Reasons are missing for 34 (3.4%), 1 (1.4%), and 5 (5.6%) emergency department visits in the not severe, progressive severe, and catastrophic severe disability groups, respectively.

[b]Grouped into distinct diagnostic categories, as described in the “Methods” section, and presented in order of highest to lowest rates in the not severe disability group.

[c]For each calculation, the numerator includes the number of emergency department visits during the follow-up period for the specific reason, while the denominator includes the number of emergency department visits for all reasons combined.

[d]Includes unclassifiable complaints such as feeling “weak,” “tired,” or generally unwell, with no other specifying features.

© 2020 Gill TM et al. *JAMA Network Open.*

eTable 3. Reasons for Restricted Activity According to Type of Severe Disability[a]

Reason for Restricted Activity[b]	Not Severe Disability (n=6205)	Progressive Severe Disability (n=185)	Catastrophic Severe Disability (n=460)
	episodes (SD) per 100 person-months [c]		
Been fatigued (no energy/very tired)	61.3 (0.5)	72.8 (0.4)	71.0 (0.5)
Pain or stiffness in joints	38.8 (0.5)	46.2 (0.5)	49.0 (0.5)
Pain or stiffness in back	30.9 (0.5)	44.0 (0.5)	41.7 (0.5)
Been dizzy or unsteady on feet	27.0 (0.4)	45.1 (0.5)	34.9 (0.5)
Cold or flu symptoms	25.3 (0.4)	19.0 (0.4)	19.6 (0.4)
Leg pain on walking	21.1 (0.4)	28.3 (0.5)	25.1 (0.4)
Been afraid of falling	20.5 (0.4)	31.0 (0.5)	27.1 (0.4)
Nausea, vomiting, diarrhea, or other stomach (abdominal) problem	19.2 (0.4)	27.2 (0.4)	15.9 (0.4)
Difficulty breathing or shortness of breath	18.6 (0.4)	30.4 (0.5)	25.7 (0.4)
Difficulty with sleeping	17.9 (0.4)	32.6 (0.5)	20.6 (0.4)
Been depressed	15.1 (0.4)	29.5 (0.5)	21.1 (0.4)
Swelling in feet or ankles	12.4 (0.3)	21.2 (0.4)	19.0 (0.4)
Been anxious or worried	12.0 (0.3)	24.6 (0.4)	19.9 (0.4)
Weakness of arms or legs	11.9 (0.3)	25.0 (0.4)	18.1 (0.4)
Poor or decreased vision	8.8 (0.3)	10.4 (0.3)	12.9 (0.3)
Change in medications	8.2 (0.3)	17.1 (0.4)	8.9 (0.3)
Fall or injury	7.6 (0.3)	10.9 (0.4)	8.7 (0.3)
Chest pain or tightness	7.0 (0.3)	11.4 (0.3)	8.7 (0.3)
Lost control of urine and wet self	6.3 (0.2)	10.4 (0.3)	6.5 (0.3)
Frequent or painful urination	5.6 (0.2)	12.0 (0.3)	7.8 (0.3)
Family member or friend became seriously ill or had an accident	4.2 (0.2)	2.7 (0.2)	5.2 (0.2)
Problem with memory or difficulty thinking	4.0 (0.2)	7.1 (0.3)	10.9 (0.3)
Experienced the death or loss of a family member or friend	3.4 (0.2)	2.7 (0.2)	3.3 (0.2)
Problem with alcohol	0	0	0

© 2020 Gill TM et al. *JAMA Network Open.*

Other reason	24.8 (0.4)	23.4 (0.4)	24.2 (0.4)

Abbreviations: SD, standard deviation

[a]The number of episodes with restricted activity are provided for each of the three disability groups. These episodes were identified from 634 (not severe disability), 75 (progressive severe disability), and 151 (catastrophic severe disability) participants.

[b]Presented in order of highest to lowest rates in the not severe disability group.

[c]For each calculation, the numerator includes the number of restricted activity episodes during the follow-up period for the specific reason, while the denominator includes the number of restricted activity episodes for all reasons combined. Because participants could provide more than 1 reason for their restricted activity, the percentages for each type of severe disability do not sum to 100.

© 2020 Gill TM et al. *JAMA Network Open.*

eTable 4. Sensitivity of Associations between Precipitants and Progressive Severe Disability to the Competing Risk of Death [a]

Imputation Approach	Hospitalization	Emergency Department Visit	Restricted Activity
	Adjusted Hazard Ratio (95% CI)[b]		
Not imputed (reported results)	48.3 (31.0–75.4)	9.6 (4.5–20.6)	3.2 (1.7–6.0)
Missing at random, censor after progressive disability [c]	33.7 (22.5–50.3)	8.5 (4.4–16.2)	2.7 (1.5–4.8)
Not missing at random, progressive disability in no months [d]	35.9 (22.9–56.3)	8.5 (3.9–18.4)	3.1 (1.7–5.9)
Not missing at random, progressive disability in last month [e]	14.7 (11.0–19.8)	4.3 (2.5–7.3)	2.8 (2.0–4.1)

Abbreviations: CI, confidence interval

[a]Follow-up was truncated by death in 113 (3.2%) of the 3550 person-intervals.

[b]Calculated using pooled logistic regression with a complementary log-log link and generalized estimating equations with robust standard errors based on an autoregressive correlation structure, as described in the Methods. The models included the same set of factors as in Figure 1.

[c]The occurrence of progressive severe disability was imputed for each month from the time of death through the end of the person-interval, with censoring at the first occurrence of the outcome for all 113 decedents.

[d]Assumes that none of the 113 decedents experienced progressive severe disability from the time of death through the end of the person-interval.

[e]Assumes that all of the 113 decedents experienced progressive severe disability only in the last month of the person-interval that was truncated by death.

© 2020 Gill TM et al. *JAMA Network Open.*

eTable 5. Sensitivity of Associations between Precipitants and Catastrophic Severe Disability to the Competing Risk of Death [a]

Imputation Approach	Hospitalization	Emergency Department Visit	Restricted Activity
	Adjusted Hazard Ratio (95% CI)[b]		
Not imputed (reported results)	321.0 (194.0–531.0)	32.8 (16.4–65.3)	10.7 (5.8–19.6)
Missing at random, censor after progressive disability [c]	206.8 (135.2–316.4)	23.2 (12.9–41.8)	6.8 (3.6–12.7)
Not missing at random, progressive disability in no months [d]	294.2 (184.6–468.7)	31.6 (16.5–60.5)	10.5 (6.0–18.4)
Not missing at random, progressive disability in last month [e]	71.2 (55.2–91.9)	9.1 (5.8–14.2)	4.6 (3.3–6.3)

Abbreviations: CI, confidence interval

[a]Follow-up was truncated by death in 113 (3.2%) of the 3550 person-intervals.

[b]Calculated using pooled logistic regression with a complementary log-log link and generalized estimating equations with robust standard errors based on an autoregressive correlation structure, as described in the Methods. The models included the same set of factors as in Figure 1.

[c]The occurrence of progressive severe disability was imputed for each month from the time of death through the end of the person-interval, with censoring at the first occurrence of the outcome for all 113 decedents.

[d]Assumes that none of the 113 decedents experienced progressive severe disability from the time of death through the end of the person-interval.

[e]Assumes that all of the 113 decedents experienced catastrophic severe disability only in the last month of the person-interval that was truncated by death.

© 2020 Gill TM et al. *JAMA Network Open.*
